# Enantioselective reduction of ketoimines promoted by easily available (*S*)-proline derivatives

**DOI:** 10.3762/bjoc.9.71

**Published:** 2013-04-02

**Authors:** Martina Bonsignore, Maurizio Benaglia, Laura Raimondi, Manuel Orlandi, Giuseppe Celentano

**Affiliations:** 1 Dipartimento di Chimica, Università degli Studi di Milano, via Golgi 19, I-20133 Milano, Italy; 2 Dipartimento di Scienze Farmaceutiche, Università degli Studi di Milano, Via Mangiagalli 25, 20133, Milano, Italy

**Keywords:** chiral prolines, imine reduction, Lewis bases, organocatalysis, trichlorosilane

## Abstract

The behavior of readily synthesized and even commercially available (*S*)-proline derivatives, was studied in the trichlorosilane-mediated reduction of ketoimines. A small library of structurally and electronically modified chiral Lewis bases was considered; such compounds were shown to promote the enantioselective reduction of different substrates in good chemical yields. In the HSiCl_3_ addition to the model substrate *N*-phenylacetophenone imine, the organocatalyst of choice led to the formation of the corresponding amine with good stereoselectivity, up to 75% ee. Theoretical studies were also performed in order to elucidate the origin of the stereoselection.

## Introduction

**Figure 1:**
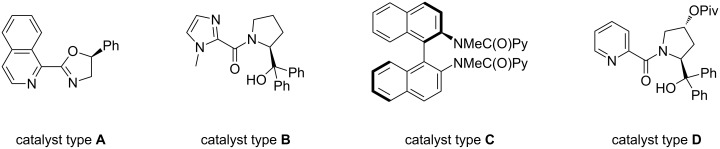
Catalysts of types **A**–**D**.

The reaction with stoichiometric amounts of trichlorosilane in the presence of a chiral catalyst is a well-established methodology to perform enantioselective reductions of carbon nitrogen double bonds [1]. In the past decade different classes of enantiomerically pure Lewis bases have been developed, including *N-*formyl derivatives, oxazolines, imidazole derivatives, sulfonamides and picolinamides [2–3]. Some of these successful catalysts essentially owe their stereodirecting ability to the steric shielding exerted by a proper spatial arrangement of the structural components (Figure 1); this is the case for example for Malkov and Kocovsky catalyst [4] **A** or Jones catalyst **B** [5–6] or the binaphthyldiamine-derived catalyst **C** developed by our group [7–8]. Also in other systems, such as the Matsumura-type catalysts developed by Zhang [9] of type **D**, the presence of a proper sterically hindered element seems to be much more decisive than the ability of the tertiary alcohol to make a possible, but not probable, weak hydrogen bond.

**Figure 2:**
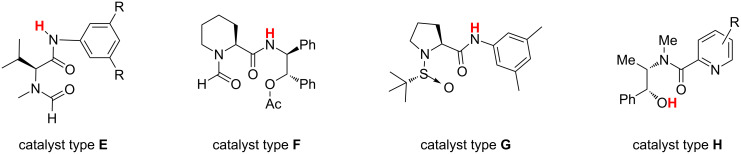
Catalysts of types **E–H**.

However, most of the more efficient systems rely on the presence of catalyst–substrate hydrogen bonding as a key structural element in controlling the stereoselectivity of the process (Figure 2). In the *N-*formyl valine derivative **E** [10–11] the proposed transition-state structure involves the formation of a hydrogen-bond between the amide group of the catalyst and the substrate as a necessary element for stereocontrol (for another example of relevant *N*-formyl proline derivative see [12]). Also in Sun catalysts **F** [13–14] and sulfinamide **G** [15] as well as in chiral picolinamides **H**, reported by Zhang [16–17] and intensely studied by our group [18–23], the presence of the acidic hydrogen of the amide group is essential for the stereochemical efficiency of the metal-free catalyst.

**Figure 3:**
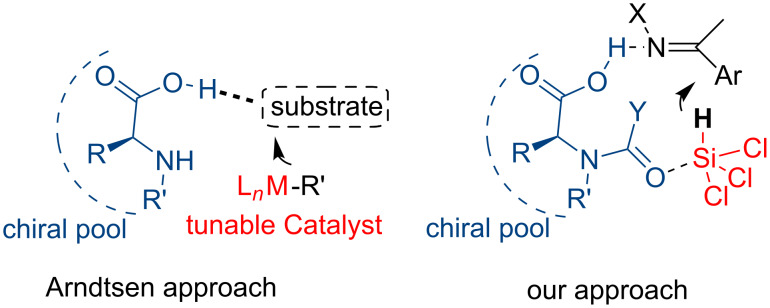
Proposed approach in this work.

Based on these considerations we decided to investigate the use of a strong hydrogen-bond donor functionality as the carboxylic acid group; it is worth mentioning that also phosphoric acid catalyzed reductions of C=N bonds with Hantzsch ester involve the coordination and activation of the substrate through the formation of a H-bond between the hydrogen atom of the phosphoric acid and the nitrogen atom of the imine [24–26]. However, we were attracted by a recent work by Arndtsen, who demonstrated that coupling metal catalysis and the ability of amino acids to form hydrogen bonds provides an easy route for inducing both enantioselectivity and selectivity (left picture in Figure 3) [27]. A simple combination of amino acid and copper catalyst provided an easily tunable system for synthesizing a range of propargylamines with high enantioselectivity. A key element of the elevate enantioselectivity was the hydrogen bonding between the chiral amino acid and the substrate, while high selectivity was achieved by tuning the metal catalyst. In the trichlorosilane-mediated reductions in this work we aimed to exploit the imine activation by the acid proton of the carboxylic group, which can act at the same time as a Lewis basic site to coordinate the silicon atom and hopefully control the stereoselectivity of the process (right picture in Figure 3).

**Scheme 1:**
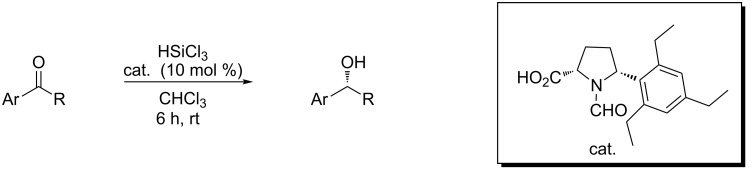
Stereoselective reduction of ketones.

It should be mentioned that Matsumura already in 2006 reported that trichlorosilane may be employed in the stereoselective reduction of ketones [28]. Catalytic amounts of *N*-formyl-α'-(2,4,6-triethylphenyl)-(*S*)-proline in combination with stoichiometric trichlorosilane allowed the formation of secondary alcohols with high levels of enantioselection (up to 97%, Scheme 1), which was strongly influenced both by the carboxylic group at the α-position and the 2,4,6-triethylphenyl group at the 5-position in the proline ring. It must be noted, however, that the successful catalyst is not an easily available compound and, in that work, no examples of carbon–nitrogen double-bond reduction were described.

## Results and Discussion

In our approach we decided to investigate very simple chiral carboxylic acids, and we turned our attention to the most obvious source of enantiopure acids, i.e., the natural amino acids. The development of readily available catalysts, synthesized in a few steps starting from inexpensive commercial sources is a long standing but still topical goal of modern asymmetric catalysis. At the beginning of this study we focused on the synthesis of very simple proline derivatives; therefore a small library of electronically and structurally different (*S*)-proline derivatives, including a few immediately available, commercial products, were easily prepared, with simple modifications at the amino acid nitrogen atom.

We decided to start our investigation testing the commercially available *N-*Boc*-*L-proline (**1**) as catalyst in the reduction of ketimines with trichlorosilane. The imines were typically prepared with a microwave-promoted reaction between acetophenone and the aromatic amine in toluene in the presence of K10 clay as activator.

The first screening allowed us to determine the best catalyst loading and the solvent of choice. Different experimental conditions and reaction temperatures were investigated. A few selected results are reported in Table 1.

**Table 1:** Enantioselective reduction with catalyst **1**.^a^

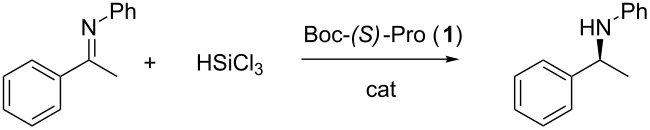						

Entry	cat. **1** (%)	solvent	*t* (h)	*T* (°C)	yield (%)^b^	ee (%)^c^

1	10	DCM	8	0	20	49
2	20	DCM	18	0	74	51
3	30	DCM	32	−20	74	73
4	30	CH_3_Cl	18	0	27	75
5	30	hexane	18	0	25	43
6	30	toluene	18	0	25	45

^a^Reaction conditions: imine (0.33 mmol), HSiCl_3_ (1.15 mmol). ^b^Yields determined after chromatographic purification. ^c^Enantiomeric excess determined by HPLC on chiral stationary phase.

These preliminary data showed that the appropriate catalyst loading seemed to be 30 mol % to achieve good yield after 18 hours. Under these conditions a modest to good level of stereoselection were observed; the best results were obtained by running the reaction in chlorinated solvents, such as dichloromethane and chloroform, where enantioselectivity up to 75% was achieved by simply performing the reduction at 0 °C.

**Figure 4:**
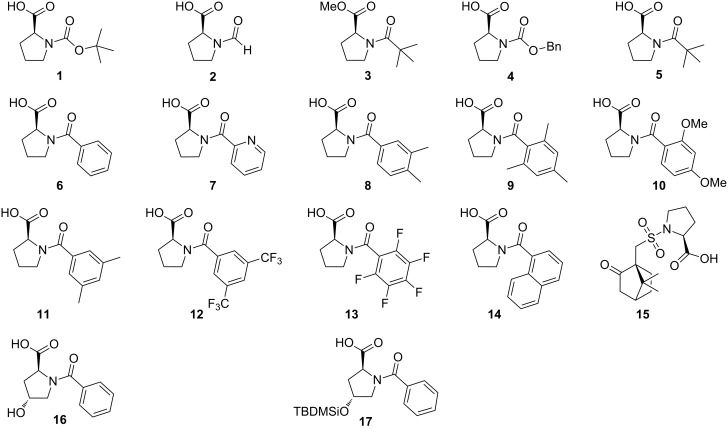
Catalysts synthesized and studied in this work.

The easy one-step reaction between (*S*)-proline and various acyl chlorides, in the presence of 1 N NaOH, allowed the formation of the catalysts illustrated in Figure 4. The acyl chlorides, when not commercially available, were prepared starting from the corresponding acid by treatment with thionyl chloride under reflux for 4 hours. The catalytic efficiency of these catalysts was evaluated in the stereoselective reduction of the *N-*phenyl imine of acetophenone (Table 2).

**Table 2:** Enantioselective reduction of the *N*-phenyl imine of acetophenone.^a^

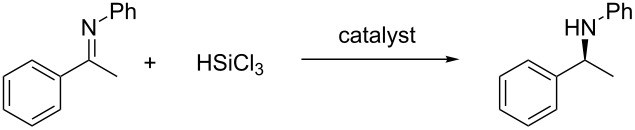			

Entry	catalyst	yield (%)^b^	ee (%)^c^

1	**1**	74	51
2	**2**	21	rac
3	**3**	49	19
4	**4**	25	49
5	**5**	36	70
6	**6**	63	59
7	**7**	98	11
8	**8**	56	30
9	**9**	54	34
10	**10**	30	27
11	**11**	74	62
12	**13**	10	8 (*R*)
13	**14**	30	32
14	**16**	26	rac
15	**17**	71	66

^a^Reaction conditions: imine (0.33 mmol), catalyst (0.09 mmol), HSiCl_3_ (1.15 mmol); 0 °C in DCM. ^b^Yields determined after chromatographic purification. ^c^Enantiomeric excess determined by HPLC on chiral stationary phase.

After running the reaction in dichloromethane at 0 °C for 18 hours, all the catalysts afforded the desired product in modest to good yields. The *N*-formyl-L-proline (**2**) led to a racemic product in 21% yield, suggesting the importance of having a bulky group on the nitrogen atom. In order to validate our hypothesis, we also tested the *N*-Boc-L-proline methyl ester (**3**): the enantiomeric excess was 19% and the yield was 49%. The outcome of this experiment strongly suggested the importance of hydrogen bonding between the catalyst and the substrate in the stereodetermining event.

By using catalysts **6** and **11** the amine was isolated with good chemical efficiency (63% and 74% yield) and discrete level of enantioselection, i.e., 59% and 62% ee, respectively. Increasing the steric hindrance on the aromatic ring did not improve the enantioselectivity and it generally lowered the chemical activity. A pyridine ring had no positive effect on the process in terms of stereocontrol (Table 2, entry 7), probably due to its coordination ability to the silicon atom, leading to multiple possible coordination modes of trichlorosilane, which are detrimental for the determination of a well-defined activation of the reducing agent and a control of the stereoselectivity.

Notably, catalyst **5**, with the pivaloyl group at the nitrogen atom, afforded the product with 36% yield and 70% enantiomeric excess. The presence of a hydroxy group on the scaffold of the catalyst (cat. **16**, Table 2, entry 14) led to a racemic product, while the silyl ether derivative (**17**, Table 2, entry 15) promoted the reaction with good enantioselectivity, showing that the hydroxy group may act as a competitive site of coordination for trichlorosilane.

Driven by these results, we focused on a more in-depth study of the most promising system. Catalysts **5** and **6** were selected to investigate the substrate scope in the enantioselective reduction of differently substituted imines (Table 3).

**Table 3:** Enantioselective reduction of differently substituted imines.^a^

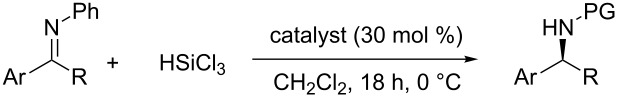						

Entry	catalyst	Ar	R	PG	yield (%)^b^	ee (%)^c^

1	**5**	Ph	CH_3_	Ph	36	70
2	**5**	Ph	CH_3_	PMP	76	68
3	**5**	4-CF_3_Ph	CH_3_	Ph	55	77
4	**5**	1-naphthyl	CH_3_	Ph	88	59
5	**5**	Ph	CH_2_CH_3_	Ph	79	5
6	**6**	Ph	CH_3_	Ph	63	59
7	**6**	Ph	CH_3_	PMP	79	55
8	**6**	4-CF_3_Ph	CH_3_	Ph	83	49
9	**6**	1-naphthyl	CH_3_	Ph	80	55
10	**6**	Ph	CH_2_CH_3_	Ph	85	53

^a^Reaction conditions: imine (0.33 mmol), catalyst (0.09 mmol), HSiCl_3_ (1.15 mmol), 0 °C, DCM. ^b^Yields determined after chromatographic purification. ^c^Enantiomeric excess determined by HPLC on chiral stationary phase.

Catalyst **5** showed a good chemical activity, promoting the enantioselective reduction in yields up to 88%, except when a very bulky protecting group was used (Table 3, entry 4). In the reaction of both *N*-Ph and *N*-PMP imines derived from acetophenone a discrete level of enantioselectivity was achieved, leading to 70% and 68% enantioselectivity, respectively. Analogously, the reduction of imine derived from 4-trifluoromethylacetophenone, led to the product in 77% ee. A remarkable drop in enantioselectivity was observed when the imine derived from propiophenone was employed (Table 3, entry 5).

Catalyst **6** followed essentially the same trend: the chemical activity was good, but no improvement of the stereochemical efficiency of the catalyst was observed. Indeed the best result was achieved on performing the reaction with the *N*-Ph imine derived from acetophenone, with 63% yield and 59% ee (Table 3, entry 6).

**Figure 5:**
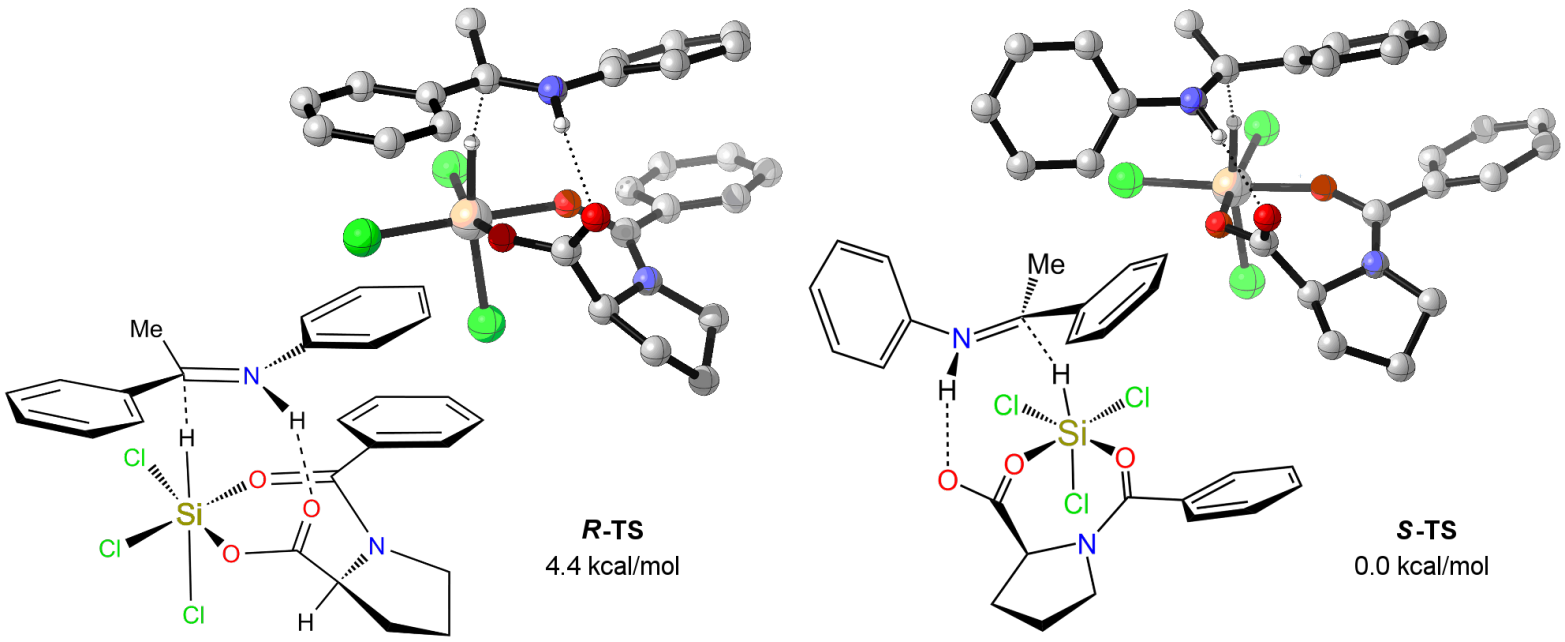
Calculated transition states for catalyst **6**.

Theoretical studies were also performed in order to elucidate the origin of the stereoselection. The reaction of *N*-phenyl imine of acetophenone with trichlorosilane promoted by catalyst **6**, i.e., the one that afforded higher yields and enantioselectivities (Table 3), was studied, and the two lowest energy transition states (TS) leading to the formation of *R* and *S* amine were located. In order to simplify the problem, we adopted a stepwise procedure for the location of the TS structures. First of all, a conformational analysis with Monte Carlo techniques was performed with MMFF [29] on a simple model of the TS, obtained by constraining the two reacting atoms (the hydride and the imine’s carboxylic carbon) at about 2.4 Å. In this way, the best arrangement for the different substituents around a model of the reaction moiety was obtained. Subsequently, the two lowest energy structures leading to the formation of the *R* and *S* products, respectively, were optimized to the relative TSs with HF/3-21G ab-initio methods [30]. After vibrational characterization of these structures (both with only one imaginary frequency), they were fully re-optimized with DFT methods [30]: optimization and calculation of the thermochemical properties was performed with M06-2X [31] functional and 6-31G basis-set (M06-2X structures are reported in Figure 5), while finer electronic energies were calculated with the 6-311G(2d,p) basis-set on the 6-31G structures. IRC calculations were also performed at the HF/3-21G level [31].

Inspection of the imaginary frequency of the two TSs, as well as IRC results, clearly indicates that the reaction, while not synchronous, is indeed concerted, since proton and hydride transfer are both occurring: while the proton transfer from the carboxylic group to the imine nitrogen is almost complete, the hydride transfer is still early, leading to a reaction classifiable as a counteranion-directed catalytic process [32]. The hydrogen bond between the proline carboxylate and the NH^+^ residues guarantees the proximity of all reactants. The calculations show the TS leading to the S-enantiomer to be more stable by 4.4 kcal/mol, which is in qualitative agreement with the experimental data.

## Conclusion

In conclusion, a series of enantiomerically pure Lewis bases directly derived from commercially available enantiopure (*S*)-proline or its derivatives was synthesized and tested in the stereoselective reduction of ketoimines in the presence of trichlorosilane. It is noteworthy that some of these catalysts are commercially available compounds and were shown to be able to promote the reaction in high chemical yield. Although the level of enantioselectivity reached in the reduction of *N-*phenylacetophenone imine was not comparable with those of the best catalyst (ee up to 75%), the low cost and the easy preparation of the chiral catalysts makes these proline-derived Lewis bases suitable candidates as starting materials for further studies and developments.

## Experimental

**General Methods.** TLC was performed on Merck silica gel 60 TLC plates F254 and visualized by using UV or phosphomolybdic acid. Flash chromatography was carried out on silica gel (230–400 mesh). ^1^H NMR spectra were recorded at 300 MHz with the indicated solvent. ^13^C NMR spectra were obtained at 75 MHz. Chemical shifts were determined relative to tetramethylsilane (for hydrogen atoms) and residual solvent peaks (for carbon atoms). Optical rotations were obtained on a Perkin-Elmer 241 polarimeter at 589 nm. HPLC for ee determination was performed on Agilent 1100 instrument under the conditions reported below. Force field conformational analysis was performed with the Schroedinger suite MacroModel. Ab initio and DFT calculations were performed with Gaussian09 [33]. Frequency calculations allowed for characterization as such of the transition structures and for calculation of ZPE-corrected free energies.

### Catalysts preparation

General procedure: (*S*)-Proline (2.7 mmol) was dissolved in 5 mL of 1 N NaOH, cooled to 0 °C in an ice–water bath and stirred magnetically. The acyl chloride (3 mmol) dissolved in THF and 2.5 mL of 1 N NaOH were added over the course of 15 min through a dropping funnel, with the temperature maintained at 5–10 °C. The pH was checked periodically to ensure that the solution remained strongly alkaline. After the addition was complete, the reaction mixture was allowed to warm to room temperature and stirred vigorously overnight. To the basic solution was added 1 N HCl until the pH became slightly acidic. Then the solution was extracted with DCM, and the organic layers were combined, dried over anhydrous MgSO_4_, concentrated on a rotary evaporator, and purified.

**Catalyst 5**. This product was purified by flash column chromatography on silica gel with 8:2 hexane/AcOEt as eluent. Yield 29%. ^1^H NMR (300 MHz, CDCl_3_) δ 4.42 (m, 1H), 3.53 (m, 1H), 1.98 (m, 2H), 1.81 (m, 2H), 1.22 (s, 9H); ^13^C NMR (75 MHz, CDCl_3_) δ 178.41 (1C), 175.62 (1C), 129.63 (1C), 61.54 (1C), 48.41 (1C), 38.91 (1C), 27.22 (3C), 26.82 (1C); [α]_D_^25^ −150.24 (*c* 0.286 g/100 mL, CHCl_3_); ESIMS *m*/*z* (%): calcd for C_10_H_17_NO_3_, 119.2; found, 119.0

**Catalyst 6**. This product was purified by flash column chromatography on silica gel with a 95:5 DCM/MeOH mixture as eluent. Yield quantitative. ^1^H NMR (200 MHz, CDCl_3_) δ 7.32 (m, 5H), 4.65 (dd, 1H), 3.53 (m, 2H), 2.00 (m, 4H); ^13^C NMR (75 MHz, CDCl_3_) δ 174.97 (1C), 170.71(1C), 135.5 (1C), 130.47 (1C), 128.27 (2C), 127.21 (2C), 59.70 (1C), 50.25 (1C), 28.93 (1C), 25.16 (1C).

### Imine reduction

General procedure: As described in [8]: to a stirred solution of catalyst (0.1–0.3 mol %) in the chosen solvent (2 mL), the imine (1 mmol) was added. The mixture was then cooled to the chosen temperature and trichlorosilane (3.5 mmol) was added dropwise by means of a syringe. After stirring at the appropriate temperature, the reaction was quenched by the addition of a saturated aqueous solution of NaHCO_3_ (1 mL). The mixture was allowed to warm up to room temperature, and water (2 mL) and dichloromethane (5 mL) were added. The organic phase was separated and the combined organic phases were dried over Na_2_SO_4_, filtered, and concentrated under vacuum at room temperature to afford the crude product. The amine was purified by flash chromatography and the absolute configuration was determined by comparison with literature data.

***N*-(Phenyl)-1-phenylethanamine.** This product was purified by flash column chromatography on silica gel with a 98:2 hexane/ethyl acetate mixture as eluent. ^1^H NMR (300 MHz, CDCl_3_) δ 7.23 (m, 7H), 6.61 (m, 3H), 4.48 (q, 1H), 1.53 (d, 3H). The enantiomeric excess was determined by HPLC on a Chiralcel OD-H (*n*-Hex/iPrOH 99:1; 0.8 mL/min; *t*_R_ (*S*) = 15.4 min; *t*_R_ (*R*) = 18.5 min).

***N*-(1-Phenylpropyl)aniline.** This product was purified with a 98:2 hexane/ethyl acetate mixture as eluent. ^1^H NMR (300 MHz, CDCl_3_) δ 7.31 (m, 4H), 7.21 (m, 1H), 7.07 (t, 2H), 6.62 (t, 1H), 6.50 (d, 2H), 4.21 (t, 1H), 4.05 (br s, 1H), 1.81 (m, 2H), 0.94 (t, 3H); HPLC: Chiralcel IB; *n*-Hex/iPrOH 99:1; 0.8 mL/min; *t*_R_ (*S*) = 7.9 min, *t*_R_ (*R*) = 8.5 min.

***N*-(1-(Naphthalen-2-yl)ethyl)aniline.** This product was purified with a hexane/ethyl acetate 98:2 mixture as eluent. ^1^H NMR (300 MHz, CDCl_3_) δ 7.89 (m, 4H), 7.5 (m, 3H), 7.1 (t, 2H), 6.7 (m, 3H), 4.6 (q, 1H), 3.97 (br s, 1H), 1.6 (d, 3H); HPLC: Chiralcel OD; *n*-Hex/iPrOH 99:1; 0.8 mL/min; *t*_R_ (*S*) = 25.2 min, *t*_R_ (*R*) = 28.4 min.

***N*-(1-(4-(Trifluoromethyl)phenyl)ethyl)aniline.** This product was purified with a 98:2 hexane/ethyl acetate mixture as eluent. ^1^H NMR (300 MHz, CDCl_3_): δ 7.51 (d, 2H), 7.41 (d, 2H), 7.02 (t, 2H), 6.59 (t, 1H), 6.38 (d, 2H), 4.46 (q, 1H), 3.97 (br s, 1H), 1.46 (d, 3H); HPLC: Chiralcel OD; *n*-Hex/iPrOH 99:1; 0.8 mL/min; *t*_R_ (*S*) = 29.3 min, *t*_R_ (*R*) = 35.7 min.

**4-Methoxy-*N*-(1-phenylethyl)aniline.** This product was purified with a 98:2 hexane/ethyl acetate mixture as eluent. ^1^H NMR (300 MHz, CDCl_3_) δ 7.43–7.26 (m, 5H), 6.73 (d, 2H), 6.58 (d, 2H), 4.46 (q, 1H), 3.74 (s, 3H), 1.58 (d, 3H); HPLC: Chiralcel IB; *n*-Hex/iPrOH 99:1; 0.8 mL/min; *t*_R_ (*S*) = 22.1 min, *t*_R_ (*R*) = 25.0 min.

***N*-Benzyl-1-phenylethanamine.** This product was purified with an 8:2 hexane/ethyl acetate mixture as eluent. ^1^H NMR (300 MHz, CDCl_3_) δ 7.38–7.24 (m, 10H), 3.82 (q, 1H), 3.67, 3.60 (AB, 2H), 1.57 (bs, 1H), 1.37 (d, 3H); HPLC: Chiralcel IB; *n*-Hex/iPrOH 99:1; 0.8 mL/min; *t*_R_ (*R*) = 8.8 min, *t*_R_ (*S*) = 9.4 min.

## Supporting Information

File 1 Synthesis and NMR spectra of catalysts, selected HPLC traces of the reduction products

## References

[1] Benaglia M, Genoni A, Bonsignore M, Rios R Enantioselective organocatalytic reductions. Stereoselective Organocatalysis.

[2] Guizzetti S, Benaglia M (2010). Eur J Org Chem.

[3] Jones S, Warner C J A (2012). Org Biomol Chem.

[4] Malkov A V, Stewart Liddon A J P, Ramirez-Lopez P, Bendova L, Haigh D, Kočovský P (2006). Angew Chem, Int Ed.

[5] Gautier F-M, Jones S, Martin S J (2009). Org Biomol Chem.

[6] Gautier F-M, Jones S, Li X, Martin S J (2011). Org Biomol Chem.

[7] Guizzetti S, Benaglia M, Celentano G (2009). Eur J Org Chem.

[8] Guizzetti S, Benaglia M, Cozzi F, Rossi S, Celentano G (2009). Chirality.

[9] Chen X, Zheng Y, Shu C, Yuan W, Liu B, Zhang X (2011). J Org Chem.

[10] Malkov A V, Mariani A, MacDougal K N, Kočovský P (2004). Org Lett.

[11] Malkov A V, Vranková K, Sigerson R C, Stončius S, Kočovský P (2009). Tetrahedron.

[12] Baudequin C, Chaturvedi D, Tsogoeva S B (2007). Eur J Org Chem.

[13] Wang Z, Ye X, Wei S, Wu P, Zhang A, Sun J (2006). Org Lett.

[14] Xiao Y-C, Wang C, Yao Y, Sun J, Chen Y-C (2011). Angew Chem, Int Ed.

[15] Wu X, Li Y, Wang C, Zhou L, Lu X, Sun J (2011). Chem–Eur J.

[16] Zheng H, Deng J, Lin W, Zhang X (2007). Tetrahedron Lett.

[17] Jiang J, Chen X, Zheng Y, Xue Z, Shu C, Yuan W, Zhang X (2011). Angew Chem, Int Ed.

[18] Guizzetti S, Benaglia M (2009). Process for the stereoselective reduction of ketoimines catalysed by trichlorosilane. WO Pat. Appl..

[19] Guizzetti S, Benaglia M, Cozzi F, Annunziata R (2009). Tetrahedron.

[20] Guizzetti S, Benaglia M, Rossi S (2009). Org Lett.

[21] Guizzetti S, Benaglia M, Biaggi C, Celentano G (2010). Synlett.

[22] Guizzetti S, Benaglia M, Bonsignore M, Raimondi L (2011). Org Biomol Chem.

[23] Bonsignore M, Benaglia M, Annunziata R, Celentano G (2011). Synlett.

[24] Akiyama T (2007). Chem Rev.

[25] Terada T (2008). Chem Commun.

[26] Yu J, Shi F, Gong L-Z (2011). Acc Chem Res.

[27] Lu Y, Johnstone T C, Arndtsen B A (2009). J Am Chem Soc.

[28] Matsumura Y, Ogura K, Kouchi Y, Iwasaki F, Onomura O (2006). Org Lett.

[29] Leach A R (2001). Molecular Modelling – principles and applications.

[30] Zhao Y, Truhlar D G (2008). Theor Chem Acc.

[31] Mahlau M, List B (2013). Angew Chem, Int Ed.

[32] (2011). MacroModel.

[33] (2009). Gaussian 09.

