# Supplementary material for: Enantioselective reduction of ketoimines promoted by easily available (S)-proline derivatives
Source: Beilstein J Org Chem. 2013 Apr 2;9:633–40. doi: 10.3762/bjoc.9.71 (PMC3628544; doi:10.3762/bjoc.9.71)
Supplement: File 1 — Synthesis and NMR spectra of catalysts, selected HPLC traces of the reduction products [file Beilstein_J_Org_Chem-09-633-s001.pdf]

Supporting Information

for

Enantioselective reduction of ketoimines promoted by easily available (*S*)-proline derivatives

Martina Bonsignore[1], Maurizio Benaglia[1,‡,*], Laura Raimondi[1], Manuel Orlandi[1] and Giuseppe Celentano[2]

Address: [1]Dipartimento di Chimica, Università degli Studi di Milano, via Golgi 19, I-20133 Milano, Italy and [2] Dipartimento di Scienze Farmaceutiche, Università degli Studi di Milano, Via Mangiagalli 25, 20133, Milano, Italy

Email: Maurizio Benaglia - maurizio.benaglia@unimi.it

‡ Tel.: +39025031-4171; Fax: +39025031-4159

*Corresponding author

Synthesis and NMR spectra of catalysts and selected HPLC traces of the reduction products

General

Besides compound **17**, all catalysts are known compounds, many of them being even commercially available products. For selected references where the molecules have been reported see, for example [1-5].

Preparation of catalyst **2**

L-proline (17.4 mmol) was dissolved in 40 mL of HCOOH (95%) and the mixture was cooled to 0 °C. Acetic anhydride (127 mmol) was added and the solution was allowed to warm to room temperature. The mixture was stirred for 2 hours at room temperature and then it was diluted with cold water (14 mL). The solvents were removed by evaporation. The product was again dissolved in *i*-PrOH (10 mL) and poured into 100 mL of Et_2O. The solvents were evaporated and the product was dried under high vacuum and obtained with 95% yield.

Two rotamers: ^{1}H-NMR (300 MHz, $CDCl_3$): δ 8.28 (s, 1H), 8.26* (s, 1H), 4.45 (m, 1H), 3.64 (m, 2H), 3.53* (t, 2H), 2.22 (m, 2H), 2.00 (m, 2H). $[\alpha]_D^{25}$ = -50.0 (solvent: CH_3OH; *c* = 5.03 g/100 mL; λ = 589 nm).

Preparation of catalysts **7**–**16** (see Experimental, General Procedure)

Catalyst **7**

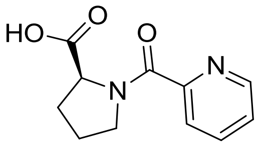

This product was purified by flash column chromatography on silica gel with a 95:5 DCM/MeOH mixture as eluent. Yield 58%; ^{1}H NMR (200 MHz, CH_3OD) δ (major) 1.71–2.24 (4H, m), 3.52–3.78 (2H, m), 4.69 (1H), 7.27–7.60 (1H, m), 7.61–7.87 (2H, m), 8.42–8.50 (1H, m); δ (minor) 1.71–2.24 (4H, m), 3.52–3.78 (2H, m), 4.41 (1H, dd), 7.27–7.60 (1H, m), 7.61–7.87 (2H, m), 8.42–8.50 (1H, m).

Catalyst **8**

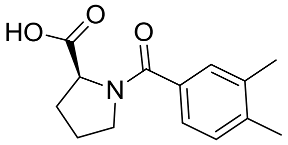

This product was purified by flash column chromatography on silica gel with a 98:2 DCM/MeOH mixture as eluent. Yield 85%; ^{1}H NMR (200 MHz, $CDCl_3$) δ 1.75 (m, 2H), 1.85 (m, 2H), 2.05 (s, 3H), 2.10 (s, 3H), 3.40 (m, 2H), 4.56 (m, 1H), 7.09 (m, 3H); ^{13}C NMR (75 MHz, $CDCl_3$) δ 174.61 (1C), 171.28 (1C), 139.35 (1C), 136.64 (1C), 133.00 (1C), 129.39 (1C), 128.53 (1C), 124.78 (1C), 59.91 (1C), 50.38 (1C), 28.65 (1C), 25.15 (1C), 19.65 (2C).

Catalyst **9**

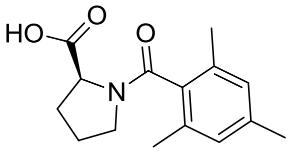

This product was purified by flash column chromatography on silica gel with a 98:2 DCM/MeOH mixture as eluent. Yield 25%; ^{1}H NMR (200 MHz, $CDCl_3$) δ 1,95 (m, 2H), 2,15 (s, 3H), 2,25 (m, 8H), 3,20 (m, 2H), 4,70 (br, 1H), 6.84 (br, 2H); ^{13}C NMR (75 MHz, $CDCl_3$) δ 177.2 (1C), 170.64 (1C), 137.89 (1C), 134.37 (2C), 132.70 (1C), 128.29 (2C), 127.80 (2C), 60.48 (1C), 48.41 (1C), 29.52 (1C), 24.89 (1C), 21.07 (1C), 18.54 (2C).

Catalyst **11**

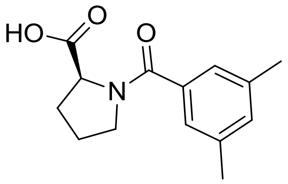

This product was purified by flash column chromatography on silica gel with a 98:2 DCM/MeOH mixture as eluent. Yield 86%; ^{1}H NMR (200 MHz, $CDCl_3$) δ 1.75 (m, 2H), 1.85 (m, 2H), 2.05 (s, 3H), 2.10 (s, 3H), 3.40 (m, 2H), 4.56 (m, 1H), 7.09 (m, 3H); ^{13}C NMR (75 MHz, $CDCl_3$) δ 174.98 (1C),

171.24 (1C), 137.97 (1C), 135.60 (1C), 131.93 (2C), 124.79 (2C), 59.45 (1C), 50.27 (1C), 28.89 (1C), 25.10 (1C), 21.37 (2C).

Catalyst **12**

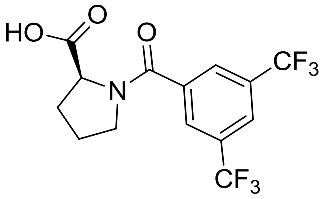

This product was purified by flash column chromatography on silica gel with a 98:2 DCM/MeOH mixture as eluent. Yield 75%; ^{1}H NMR (200 MHz, $CDCl_3$) δ 2.15 (m, 4H), 3.55 (m, 2H), 4.78 (dd, 1H), 7.96 (s, 1H), 8.04 (s, 2H).

Catalyst **13**

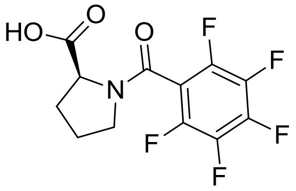

This product was purified by flash column chromatography on silica gel with a 98:2 DCM/MeOH mixture as eluent. Yield 75%; ^{1}H NMR (200 MHz, $CDCl_3$) δ 2.05 (m, 2H), 2.24 (m, 1H), 2.36 (m, 1H), 3.47 (m, 2H), 4.68 (dd, 1H).

Catalyst **14**

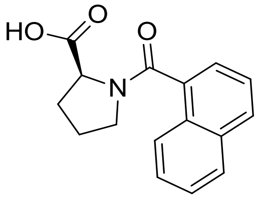

This product was purified by flash column chromatography on silica gel with a 98:2 DCM/MeOH mixture as eluent. Yield 27%; ^{1}H NMR (200 MHz, CD_3OD) δ 1.90 (m, 2H), 2.10 (m, 2H), 2.42 (m, 1H), 3.23 (m, 1H), 4.71 (m, 1H), 7.56 (m, 4H), 7.92 (m, 2H), 8.12 (m, 1H).

Catalyst **15**

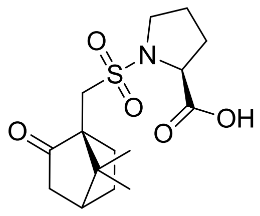

This product was purified by flash column chromatography on silica gel with a 98:2 DCM/MeOH mixture as eluent. Yield 22%; ^{1}H NMR (200 MHz, $CDCl_3$) δ 0.84 (s, 3H), 1.06 (s, 3H), 1.24 (m, 1H), 1.40 (m, 1H), 1.64 (m, 1H), 1.93 (d, 1H), 2.05 (m, 4H), 2.39 (m, 3H), 3.08 (d, 1H), 3.56 (m, 3H), 4.52 (dd, 1H).

Catalyst **16**

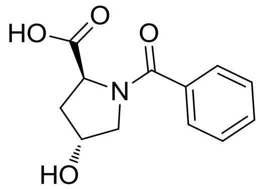

This product was purified by flash column chromatography on silica gel with a 8:2 DCM/MeOH mixture as eluent. Quantitative yield; ^{1}H NMR (300 MHz, MeOD) δ 7.50 (m, 5H), 4.88 (s, 2H), 4.72 (t, 1H), 4.40 (s, 1H), 3.82 (d, 1H), 3.45 (d, 1H), 2.43 (m, 1H), 2.20 (m, 1H); ^{13}C NMR (75 MHz, MeOD) δ 175.50 (1C), 171.01 (1C), 135.80 (1C), 132.08–126.80 (5C), 69.70 (1C), 58.80 (1C), 58.02 (1C), 37.50 (1C); $[\alpha]_D^{25}$ −111.2 (*c* 0.258 g/100mL, MeOH); ESIMS *m*/*z* (%): calcd for $C_{12}H_{13}NO_4$, 235.24; found, 235.0.

Catalyst **17**

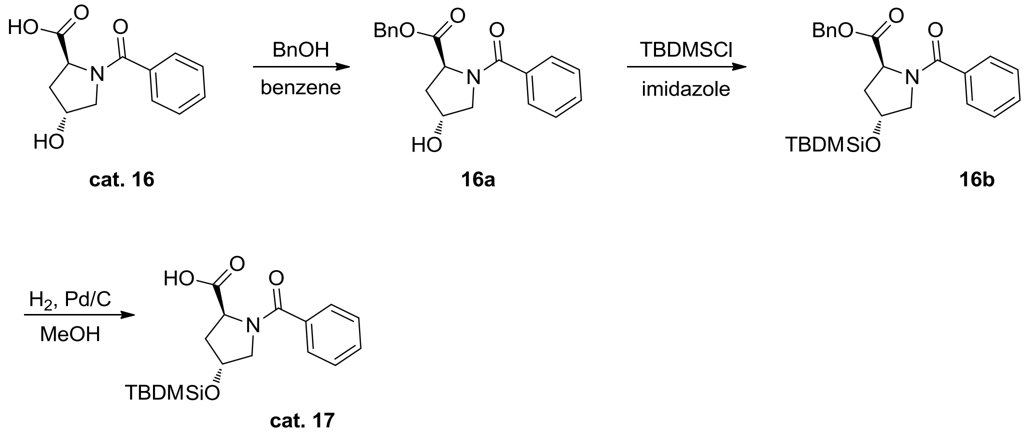

16a. A stirred solution of catalyst **16** (7.6 mmol) in benzene (6 mL) and benzyl alcohol (6 mL) containing *p*-toluensulfonic acid (7.6 mmol) was heated under reflux overnight. Solvents were removed by distillation and the crude product was purified by flash chromatography (7:3 Hex/AcOEt) to give **16a** in quantitative yield. ^{1}H NMR (300 MHz, $CDCl_3$) δ 7.49 (m, 2H), 7.33 (m, 8H), 5.60 (m, 2H), 4.83 (m, 1H), 4.34 (br, 1H), 3.67 (m, 1H), 3.41 (m, 1H), 2.32 (m, 1H), 1.99 (m, 1H).

16b. To a solution of **16a** (0.58 mmol) and imidazole (0.70 mmol) in THF (4 mL) at 0 °C, TBDMSiCl (0.70 mmol) was added dropwise. The mixture was allowed to warm to room temperature and stirred for 18 h. The solution was filtered and concentrated in vacuo and the product was used in the next step without further purification. ^{1}H NMR (300 MHz, $CDCl_3$) δ 7.55–7.25 (m, 10H), 5.23 (s, 2H), 4.85 (m, 1H), 4.39 (br, 1H), 3.70 (m, 1H), 3.36 (m, 1H), 2.27 (m, 1H), 2.06 (m, 1H), 0.80 (s, 9H), −0.04 (s, 3H), −0.06 (s, 3H).

Catalyst 17. A suspension of **16b** (0.58 mmol) and Pd/C (0.06 mmol) in methanol (3.5 mL) was stirred under a hydrogen atmosphere at room temperature for 5 h. The catalyst was removed by filtration through a celite pad, and the filtrate was concentrated to afford **17** in quantitative yield. ^{1}H NMR (300 MHz, $CDCl_3$) δ 7.51–7.33 (m, 5H), 4.77 (m, 1H), 4.35 (br, 1H), 3.55 (m, 1H), 3.37 (m, 2H), 2.21 (m, 2H), 0.79 (s, 9H), 0.00 (s, 3H), −0.08 (s, 3H); ^{13}C NMR (75 MHz, $CDCl_3$) δ 175.04 (1C), 172.09 (1C), 135.75 (1C), 130.82 (1C), 128.68 (2C), 127.71 (2C), 70.79 (1C), 59.14 (1C), 58.79 (1C), 38.03 (2C), 25.94 (3C), 4.31 (2C); $[\alpha]_D^{25}$ −67.2 (*c* 0.15 g/100mL, CH_2Cl_2); ESIMS *m/z* (%): calcd for $C_{18}H_{27}NO_4Si$, 349.17; found, 349.11.

Synthesis of imines

General procedure: Amine (1 equiv) was reacted in toluene with ketone (1 equiv) in the presence of montmorillonite (250 mg for 5 mmol of reagent) in a microwave reactor (PW = 200 W; T = 130 °C; time: 4.5 h). The product was purified by fractional distillation at p = 1 mbar.

N-phenyl-(1-phenylethylidene)amine

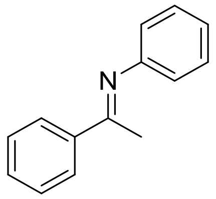

Yield: 70%; ^{1}H NMR (300 MHz, $CDCl_3$) δ 8.00–7.95 (m, 2H), 7.49–7.41 (m, 3H), 7.39–7.32 (m, 2H), 7.12–7.06 (m, 1H), 6.83–6.77 (m, 2 H), 2.24 (s, 3H).

N-Phenyl-(1-phenylpropylidene)amine

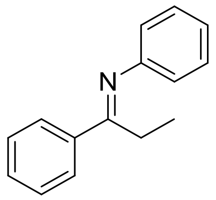

Yield: 76%; ^{1}H NMR (300 MHz, $CDCl_3$) δ major isomer: 7.95–7.91 (m, 2H), 7.48–7.43 (m, 3H), 7.37–7.31 (m, 2H), 7.11–7.04 (m, 1 H), 6.81–6.77 (m, 2H), 2.66 (q, 2H), 1.08 (t, 3H); minor isomer: 2.80 (q, 2H), 1.23 (t, 3H).

N-Phenyl-[1-(2-naphthyl)ethylidene]amine

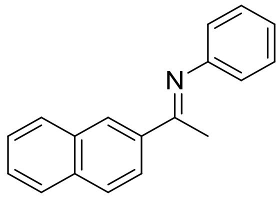

Yield: 80%; ^{1}H NMR (300 MHz, $CDCl_3$) δ 8.35 (s, 1H), 8.23 (m, 1H), 7.96–7.86 (m, 3H), 7.58–7.50 (m, 2H), 7.41–7.35 (m, 2H), 7.11 (m, 1H), 6.85 (m, 2H), 2.36 (s, 3H).

N-(1-(4-(Trifluoromethyl)phenyl)ethylidene)aniline

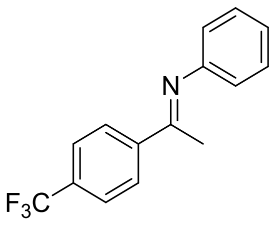

Purification: crystallization from hexane; yield: 40%; ^{1}H NMR (300 MHz, $CDCl_3$) δ 8.06 (d, 2H), 7.70 (d, 2H), 7.4–7.1 (m, 3H), 6.8 (d, 2H), 2.26 (s, 3H).

4-Methoxy-*N*-(1-phenylethylidene)aniline

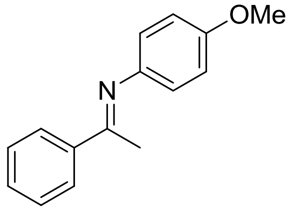

Yield: 80%; ^{1}H NMR (300 MHz, $CDCl_3$) δ 7.96 (m, 2H), 7.45 (m, 3H), 6.92 (d, 2H), 6.74 (d, 2H), 3.81 (s, 3H), 2.25 (s, 3H).

N-Benzyl-(1-phenylethylidene)amine

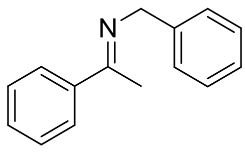

Yield: 60%; ^{1}H NMR (300 MHz, $CDCl_3$) δ major isomer: 7.90–7.88 (m, 2H), 7.46–7.27 (m, 8H), 4.46 (s, 2H), 2.35 (s, 3H); minor isomer: 4.44 (s, 2H), 2.40 (s, 3H).

***N*-(Phenyl)-1-phenyl-ethanamine**

Sample Info EP14
CHIRALCEL OD HEX:IPA 99:1, 0.8 ml/min 25bar

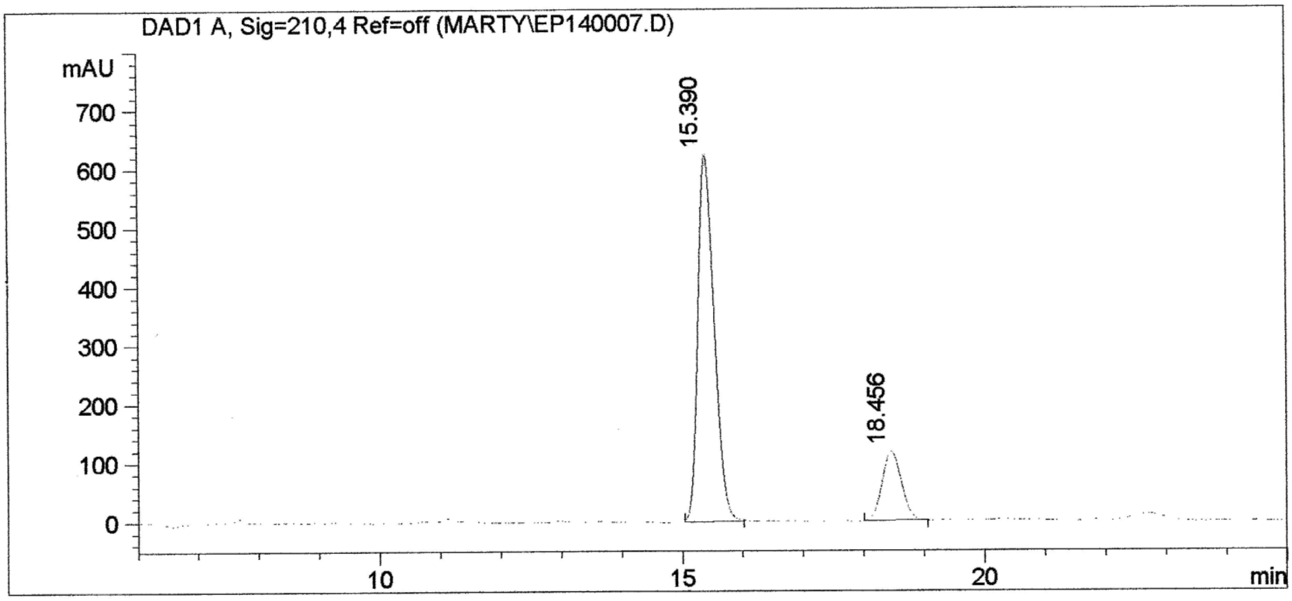

***N*-(1-(Naphthalen-2-yl)ethyl)aniline**

Sample Info MA5COPH
CHIRALCEL OD HEX/IPA 99:1, 0.8ml/mil P=9bar

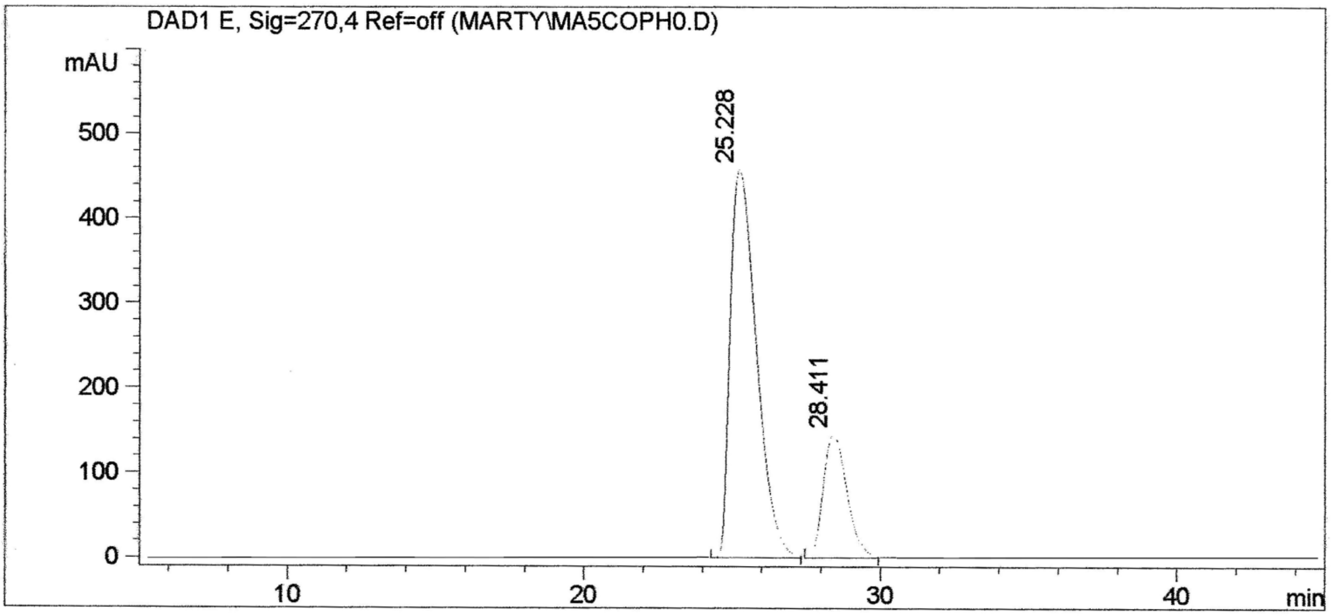

N-(1-(4-(Trifluoromethyl)phenyl)ethyl)aniline

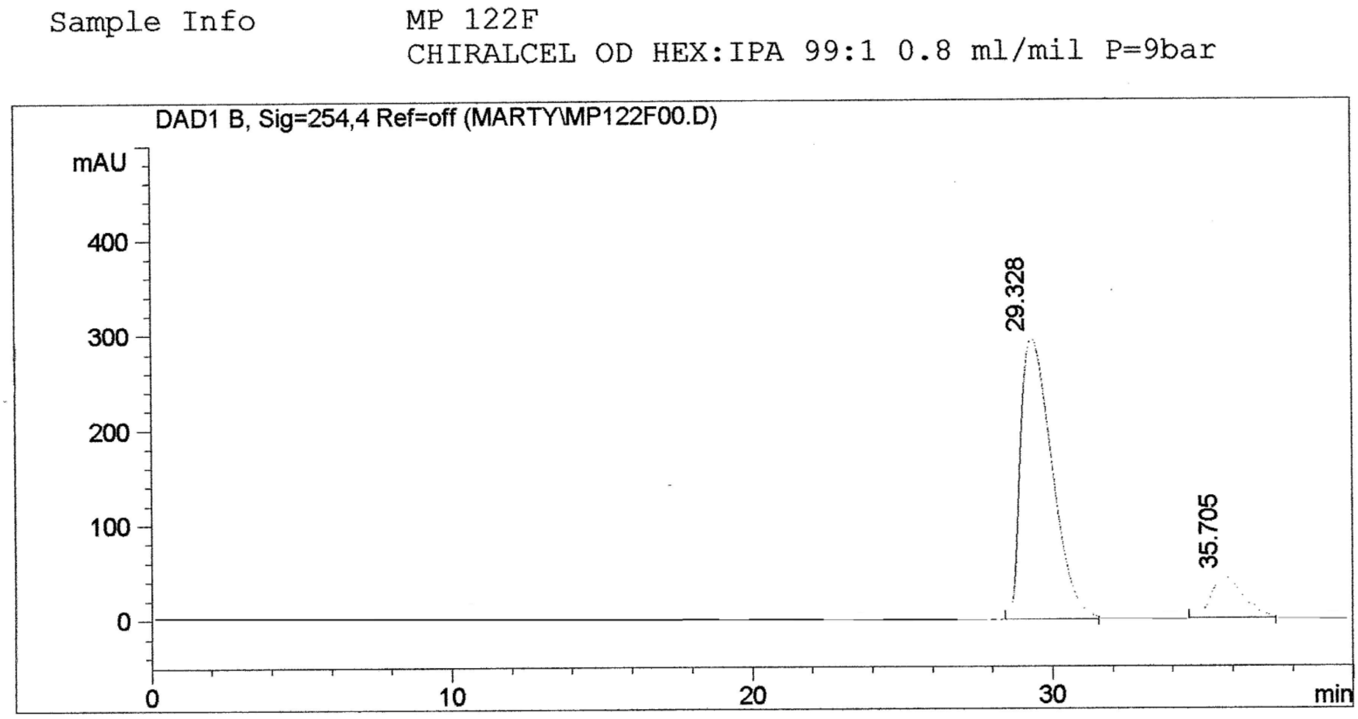

References

1. C. Coors, R. Matusch, *Journal of High Resolution Chromatography and Chromatography Communications* (1988), 11(5), 422-3
2. M. Kawase, H. Miyamae, T. Kurihara, *Chemical & Pharmaceutical Bulletin* (1998), 46(5), 749-756.
3. K. Yoshikawa, Kiyoshi, K. Achiwa, *Chemical & Pharmaceutical Bulletin* (1995), 43(12), 2048-53.
4. T. González, O. Abad, M. Santano, C. Minguillón, *Synthesis*, (2004), 1171.
5. S. B. Matin, M. Rowland, N. Castagnoli Jr., *J. Pharmaceutical Science* (1973), 821.
